# Advanced Oxidation Protein Products and Carbonylated Proteins as Biomarkers of Oxidative Stress in Selected Atherosclerosis-Mediated Diseases

**DOI:** 10.1155/2017/4975264

**Published:** 2017-08-13

**Authors:** Bogna Gryszczyńska, Dorota Formanowicz, Magdalena Budzyń, Maria Wanic-Kossowska, Elżbieta Pawliczak, Piotr Formanowicz, Wacław Majewski, Krzysztof Wojciech Strzyżewski, Magdalena P. Kasprzak, Maria Iskra

**Affiliations:** ^1^Department of General Chemistry, Chair of Chemistry and Clinical Biochemistry, Poznan University of Medical Sciences, Grunwaldzka 6, 60-780 Poznan, Poland; ^2^Department of Clinical Biochemistry and Laboratory Medicine, Chair of Chemistry and Clinical Biochemistry, Poznan University of Medical Sciences, Grunwaldzka 6, 60-780 Poznan, Poland; ^3^Department of Nephrology, Transplantology and Internal Medicine, Poznan University of Medical Sciences, Przybyszewskiego 49, 60-355 Poznan, Poland; ^4^Institute of Computing Science, Poznan University of Technology, Piotrowo 2, 60-965 Poznan, Poland; ^5^Institute of Bioorganic Chemistry, Polish Academy of Sciences, Noskowskiego 12/14, 61-704 Poznan, Poland; ^6^Department of General and Vascular Surgery, Poznan University of Medical Sciences, Dluga 1/2, 61-848 Poznan, Poland

## Abstract

**Objectives:**

The main question of this study was to evaluate the intensity of oxidative protein modification shown as advanced oxidation protein products (AOPP) and carbonylated proteins, expressed as protein carbonyl content (C=O) in abdominal aortic aneurysms (AAA), aortoiliac occlusive disease (AIOD), and chronic kidney disease (CKD).

**Design and Methods:**

The study was carried out in a group of 35 AAA patients and 13 AIOD patients. However, CKD patients were divided into two groups: predialysis (PRE) included 50 patients or hemodialysis (HD) consisted of 34 patients. AOPP and C=O were measured using colorimetric assay kit, while C-reactive protein concentration was measured by high-sensitivity assay (hsCRP).

**Results:**

The concentration of AOPP in both AAA and AIOD groups was higher than in PRE and HD groups according to descending order: AAA~AIOD > HD > PRE. The content of C=O was higher in the PRE group in comparison to AIOD and AAA according to the descending order: PRE~HD > AAA~AIOD.

**Conclusions:**

AAA, AIOD, and CKD-related atherosclerosis (PRE and HD) contribute to the changes in the formation of AOPP and C=O. They may promote modification of proteins in a different way, probably due to the various factors that influence oxidative stress here.

## 1. Introduction

The oxidative modification of proteins, as a result of oxidative stress, is an irreversible process, leading to pathological conditions of the vascular system. However, the harmful effect of such modifications has been so far poorly investigated in AAA, AIOD, and CKD where atherosclerosis is crucial underlying process. Oxidative stress occurs locally in AAA, more systemic due to lumen narrowing or closing of distal part of the abdominal aorta in AIOD [1], whereas in CKD it is either local or systemic.

AOPP and C=O are well-known biomarkers of the oxidative modification of proteins. AOPP are formed in the reaction of chlorinated oxidants, such as chloramines and hypochlorous acid (HOCl), with plasma proteins, and act as the most commonly measured marker of oxidative stress in many diseases [2]. They are most frequently derived from plasma albumins and, interestingly, the chromatographic analysis of uremic plasma distinguished between high-molecular weight and low-molecular weight AOPP (monomeric form of albumin) [2]. Nevertheless, AOPP should be recognized as one of a general biomarkers of oxidative stress because their molecular composition have been so far poorly classified [2, 3]. This group of modified proteins includes dityrosine (di-Tyr), pentosidine, and carbonyl-containing protein products (reactive C=O) [3, 4]. Furthermore, it probably influences reproducibility and accuracy of spectrophotometric measurement of their concentration based on reaction with chloramine T at 340 nm [5]. In this respect, it is difficult to interpret which “subgroup” of AOPP is in fact detected on the basis of colorimetric method. Consequently, the levels of AOPP in various diseased are often contrary and reference values of healthy subject are not available in literature.

**Figure 1:**
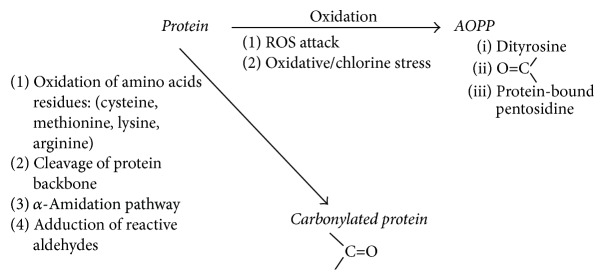
Major pathways leading to formation of AOPP and C=O. AOPP are group of oxidatively modified protein products containing dityrosine, pentosidine, and carbonyl-containing products generated by ROS or formed via myeloperoxidase reaction during oxidative/chlorine stress. Carbonylation of proteins, which is the other mechanism of protein modification, may be due to a direct oxidation of amino acids residues by ROS or nonoxidative reactions with carbonyl-containing oxidized lipids. Cleavage of proteins backbone and *α*-amidation are recognized as other pathways leading to C=O. Finally, reactive aldehydes, which are classified as main products of metal-catalysed oxidation of unsaturated fatty acids, are attached to protein chain.

Carbonylation of proteins, which is the other mechanism of protein modification, may be due to a direct oxidation of lysine, arginine, proline, and threonine residues or through interaction with reactive oxygen species (ROS) and nonoxidative reactions with carbonyl-containing oxidized lipids [5]. Cleavage of proteins, *α*-amidation, and oxidation of glutamine side chains are recognized as other pathways leading to C=O [6, 7]. The major pathways leading to formation of AOPP and C=O are summarized in Figure 1. The measurement of C=O is used to analyze the intensity of oxidative stress at the same frequency as AOPP. It should be noted that, similar to AOPP assay, spectrophotometric measurement of C=O concentration based on reaction with 2,4-dinitrophenylhydrazine is also not specific.

The aim of the study was to establish effect of AAA, AIOD, and CKD on the selected oxidative modification of proteins. AOPP and C=O have been chosen as representative biomarkers of proteins modification in studied atherosclerosis-mediated diseases.

## 2. Material and Methods

### 2.1. Patients

The study protocol conforms to the ethical guidelines of the World Medical Association Declaration of Helsinki. All study participants fulfilled the study criteria and completed the study. Before the project commenced, appropriate approval had been obtained from the Bioethical Commission of the Karol Marcinkowski University of Medical Sciences (number 854/14). All patients qualified for this study underwent a careful interview and a clinical examination with an evaluation of patients' history based on the hospital and outpatient records.

#### 2.1.1. AAA and AIOD Patients

The study was carried out in a group of 35 AAA patients (26 men and 9 women; mean age 69.97 ± 9.97) and AIOD group of 13 patients (9 men and 4 women; mean age 63.54 ± 7.55), who had been admitted to the Department of General and Vascular Surgery at the University of Medical Sciences, Poznan, Poland. In all AAA and AIOD patients, Doppler ultrasonography, computed tomography, or arteriography was conducted. The mean internal diameter of aneurysms in AAA patients was 59.88 ± 11.71 mm.

#### 2.1.2. CKD Patients

Patients treated in the Department of Nephrology, Transplantology and Internal Medicine, Poznan University of Medical Sciences, were assigned to one of the two CKD groups, depending on the severity of CKD, that is, either predialysis patients (PRE) or hemodialysis patients (HD). The identification and staging of CKD were based on the measurement of estimated glomerular filtration rate (eGFR). There are five stages of CKD used in medical practice, ranging from 1 to 5, where 5 describes the most advanced stage of CKD, usually treated by dialysis. The first CKD group, named PRE (stages 3-4), included patients (*n* = 50) with a severely reduced kidney function (eGFR 24.53 ± 9.35 ml/min/1.73 m^2^). The second CKD group, called HD group (stage 5), consisted of 34 patients with a very severely reduced kidney function, who have been treated with maintenance HD for at least 12 months (eGFR 7.29 ± 3.17 ml/min/1.73 m^2^).

#### 2.1.3. The Exclusion Criteria for All of the Groups

The exclusion criteria were the following: diabetes mellitus, albuminuria (urinary albumin to creatinine ratio ≥ 30 mg/g), current or recent (<1 month) active acute infection, immune-suppressive treatment, kidney transplantation, abnormal liver function, malignant tumors in the past 5 years, smoking, and/or alcohol abuse in the past 5 years.

The clinical data (particularly regarding cardiovascular risk factors) including the information on the diagnosed concomitant diseases and the medications taken by particular patients are summarized in Table 1, whereas Table 2 presents biochemical characteristics of all patients qualified for the study.

### 2.2. Sample Collection

Blood samples were drawn from the arms of AAA, AIOD, PRE, and HD patients. Blood samples were taken from subjects in the recumbent position after 10 minutes of rest. Samples were collected into heparin anticoagulant tubes. Next, the tubes were centrifuged at 3.000 rpm for 15 minutes. Plasma samples were stored at the temperature of −80°C until all of assays were performed. For all CKD patients, blood samples were collected at the time of the standard monitoring blood tests. In the case of HD group, blood samples were always drawn before the second HD session of the week.

### 2.3. Laboratory Analysis

#### 2.3.1. Biochemical Parameters

Medonic M20 automatic analyzer (Clinical Diagnostic Solution, Inc., USA) was used to determine the following parameters: white blood cells count (WBC), red blood cells count (RBC), hemoglobin (HGB), hematocrit (HCT), and blood plates (PLT). Blood biochemical analysis was performed using EasyRA analyzer (Medica, USA) and included the determination of uric acid (UA), albumin, total cholesterol (TC), triglycerides (TAG), LDL-cholesterol (LDL-C), HDL-cholesterol (HDL-C), fibrinogen (FB), creatinine (Cr), and urea (U).

#### 2.3.2. Total Protein Content, AOPP, Protein Carbonyl Content, and hsCRP

Enzyme-linked-immunosorbent assay or colorimetric assay kit was carried out using Zenyth 200 Microplate Spectrophotometer (Anthos Labtec Instruments GmbH).

*(1) Total Protein Content*. The protein content was determined using Protein Carbonyl Colorimetric Assay Kit (Cayman Chemical Company, USA). Proteins, which are lost during the washing steps, are diluted with guanidine hydrochloride. Finally, the absorbance is measured spectrophotometrically at 280 nm. The concentration of protein is calculated based on a bovine serum albumin standard curve.

*(2) AOPP*. AOPP Assay Kit (Cell Biolabs, Inc., USA) is based on the reaction between chloramine reaction initiator and AOPP in unknown AOPP-containing samples. The latter are first mixed with the reaction initiator which starts a color development process. After incubation, a stop solution is added. The AOPP content is determined through comparison with the predetermined chloramine standard curve by reading the absorbance on a spectrophotometric plate reader using 340 nm as the primary wave length. The concentration of AOPP is expressed per total protein content [*μ*mol/mg protein].

*(3) Protein Carbonyl Content*. Protein Carbonyl Colorimetric Assay Kit (Cayman Chemical Company, USA) is based on the reaction between 2,4-dinitrophenylhydrazine (DNPH) and protein carbonyls forming Schiff base. Eventually, the reaction leads to the formation of hydrazine, which then can be analyzed spectrophotometrically at *λ* = 360–385 nm. The concentration of C=O is expressed per total protein content [nmol/mg protein].

*(4) hsCRP*. The hsCRP determination was based on ELISA assay (DRG International, Inc., USA). The CRP molecules present in an analyzed sample are sandwiched between the solid phase and enzyme-linked antibodies. 45-minute incubation at room temperature is required after which the wells should be washed. Next, tetramethylbenzidine (TMB) is added and incubated for 20 minutes. The blue complex precipitates and the color development are stopped by adding 1 N HCl, which results in the formation yellow color. Finally, the absorbance is measured spectrophotometrically at 450 nm and the concentration of CRP is proportional to the color intensity of the analyzed sample. The lower limit of hsCRP is *≅*0.1 mg/L whereas the upper limit is 10 mg/L CRP.

### 2.4. Statistical Analysis

The statistical analysis was conducted using GraphPad Prism software 5.0 (GraphPad Software, San Diego, CA). The Kolmogorov-Smirnov or Shapiro-Wilk test was used to test the normality of quantitative variables. Normally distributed, continuous variables were analyzed using the Student's *t*-test. The Pearson or the Spearman correlation coefficient was used to test the strength of any associations between different variables. In all cases, *p* value ≤ 0.05 was considered significant.

## 3. Results

**Figure 2:**
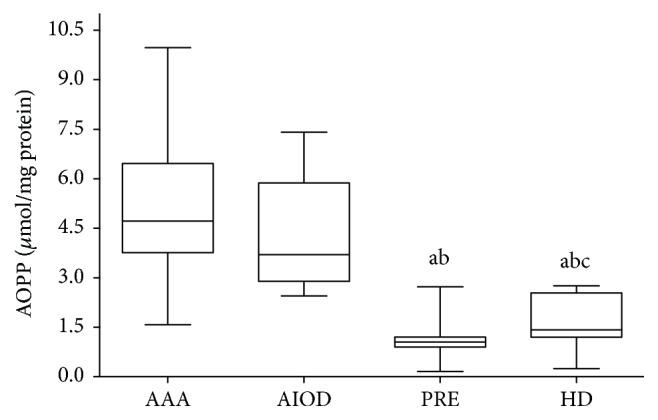
Concentrations of AOPP in studied groups expressed as *μ*mol/mg protein. Box and whisker plots show median (central line), upper, and lower quartiles (box) and range excluding outliers (whiskers); ^a^significant differences versus AAA, ^b^significant differences versus AIOD, and ^c^significant differences versus PRE *p* ≤ 0.05.

AOPP and C=O levels were found to be different among studied group of patients. The data presented in Figure 2 show higher concentration of AOPP in AAA group and AIOD group in comparison with PRE and HD groups.

**Figure 3:**
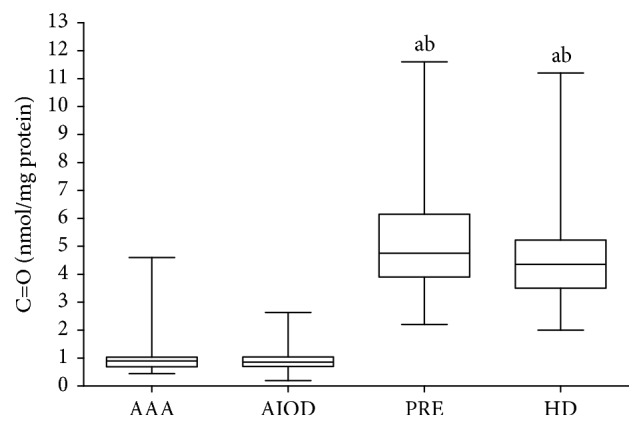
Concentration of C=O in studied groups. Box and whisker plots show median (central line), upper, and lower quartiles (box) and range excluding outliers (whiskers); ^a^significant differences versus AAA and ^b^significant differences versus AIOD *p* ≤ 0.05.

The median concentration of AOPP was as follows: 4.72 *μ*mol/mg protein (range: 1.58–9.97) in the AAA group, 3.70 *μ*mol/mg protein (range: 2.45–7.41) in the AIOD group, 1.05 *μ*mol/mg protein (range: 0.16–2.72) in the PRE group, and 1.42 *μ*mol/mg protein (range: 0.25–2.76) in the HD group, respectively. A significantly higher concentration of AOPP in the AAA group was observed in comparison with PRE and HD groups and lower in the PRE group than in the HD group in the descending order: AAA~AIOD > HD > PRE. The median values of C=O were the following: 0.89 nmol/mg protein (range: 0.44–4.60) in the AAA group; 0.86 nmol/mg protein (range: 0.19–2.63) in the AIOD group; 4.35 nmol/mg protein (range: 2.00–11.20) in the HD group; and 4.75 nmol/mg protein (range: 2.20–11.60) in the PRE group, respectively. The content of C=O was higher in the PRE group in comparison to AIOD and AAA according to the descending order: PRE~HD > AAA~AIOD (Figure 3).

Positive and significant correlations were found in the AAA group for AOPP and TC, LDL, TG, and FB as well as for C=O and UA, respectively. The negative correlation for C=O and eGFR in AAA was found (Table 3). In AIOD group, AOPP concentration was found significantly correlated both with C=O and U concentration. The statistical analysis of the results obtained for PRE patients shown a positive and significant correlation between C=O and HCT and a negative correlation for C=O. Moreover, a positive and significant correlation was found in the HD group for C=O and WBC. Correlations coefficients in the analyzed groups are presented in Table 3. AOPP and C=O were analyzed as categorical variables. All patients of the examined groups were divided into three subgroups using 25th and 75th percentiles (group I < 25th percentile, groups II and III 25th–75th percentile, and group IV > 75th percentile) based on AOPP and C=O levels (Table 4). Division of the studied groups into quartiles according to AOPP and C=O concentration differences between studied parameters were revealed. The data presented in Table 4 demonstrate significant differences in TC, TAG, and FB values between quartiles in AAA group. Significant differences between C=O and U values in comparison with quartile I were shown in the AIOD group. WBC count and eGFR were found to be different between quartiles in PRE group. Comparison quartiles of C=O concentration demonstrated a significant difference in AOPP concentration in quartile I versus quartile IV between AIOD and HD patients. Significant differences between AOPP and eGFR values between quartiles were shown in the HD group.

## 4. Discussion

Atherosclerosis is a multiple inflammatory-proliferative process caused by various factors. It involves series of pathological processes affecting cardiovascular system, immune system, and lipids metabolism. In relation to complex etiology of atherosclerosis, the studies on its pathomechanism are still essential and should be continued. Several reports have indicated that two types of atherosclerosis are defined: “classic” associated with cardiovascular disease (CVD) and “nonclassic” CKD [8, 9]. The above-mentioned classification is based on differing mechanisms underlying the formation of CVD- and CKD-related plaques [10]. In this respect, patients with “classic” atherosclerosis (AAA and AIOD) and “nonclassic” (PRE and HD) atherosclerosis were qualified for the present study. Although no significant differences in concentration of TC, LDL-C, LDL-C, and TAG between AAA, AIOD, PRE, and HD patients have been detected, they are characterized as having a high cardiovascular risk (their hsCRP was between 3–10 mg/l, depending on the group). Furthermore, based on literature data, we can assume that oxidative stress associated with “classic” and “nonclassic” atherosclerosis is different. In this context, the main objective of this study was to determine the intensity of oxidative modification of proteins expressed as a level of AOPP and C=O in selected diseases which are associated with atherosclerosis.

In the present study, concentration of AOPP in blood plasma was found higher among both in AOID and in AAA patients in comparison to PRE and HD patients. However, protein carbonylation was more intense in PRE and HD than in AOID and AAA group. Two possible explanations are proposed. The first one assumes that “classic atherosclerosis” and CKD-related atherosclerosis and, consequently, associated oxidative stress impact proteins in different way. The other explanation is that spectrophotometric measurement of AOPP and C=O concentration is not specific.

It is clear that presence of plasma AOPP and C=O in AAA, AIOD, and CKD patients indicates the effects of oxidative stress to plasma proteins. Classical oxidative stress and carbonyl, nitrosative, and chlorine stress were widely studied in many diseases [4, 11–13]. The pathogenic effect of oxidative stress involves enhancing of proatherosclerosis mechanisms and cardiovascular risk in patients with CKD [14]. Increased mitochondrial production of ROS may activate proinflammatory pathways and affect the structure of lipids and proteins and enzyme activity [15]. Although four different pathways of oxidative stress probably take place in CKD, literature data suggest that nitrosative and carbonyl stress plays a crucial role here [11, 16]. Therefore, uremia may be characterized as carbonyl overload or “carbonyl stress” which reflects increasing oxidation of proteins, carbohydrates, and lipids and no effective detoxification or complications associated with chronic renal failure and dialysis [17, 18].

Oxidative stress may link multiple mechanisms of AAA including hypertension, vascular inflammation, and increased activity of metalloproteinases [19, 20]. Currently, in more than 90% of AAA cases, which are located below the renal arteries, aneurysm formation is associated with atherosclerotic process [21]. Oxidative protein modification is one of the processes intensifying oxidative stress and pathological damage to the arterial wall. Based on literature data, it is difficult to conclude which pathway of oxidative stress dominates in AAA and AIOD. Clinical observations indicate that oxidative stress occurs locally in AAA but is more systemic in AIOD [1, 20, 22].

It is well-known that different oxidation reactions proteins may take place simultaneously [23, 24]. As a result, a protein molecule may be oxidized with different intensity, at several amino acids side chains, and its mass increases depending on type of modification [23, 24]. In that connection, identification and chemical characterization of modified protein are, certainly, crucial for analysis of modified proteins.

Various analytical methods have been described for qualitative and quantitative analysis of C=O, that is, from derivatization of C=O with DNPH, to reversed phase chromatography and mass spectrometry [25, 26]. Interestingly, Mirzaei et al. concluded that probably not all carbonylated sites of protein are identified using advanced analytical methods, such as chromatography and tandem mass spectrometry [27]. Based on the literature and our results, we may assume that spectrophotometric measurement of C=O concentration based on reaction with DNPH applied in the present study does not detect probably all carbonylated proteins. DNPH reacts with “free” reactive C=O groups only, while less available C=O groups or highly modified carbonylated proteins do not react. Similar assumptions were made for spectrophotometric measurement of AOPP based on reaction with chloramine T. Although their composition has not yet been accurately characterized, in its structure dityrosine, C=O and cross-linking bonds are predominate [28]. Therefore, it is difficult to determine which type of modification is directly detected with chloramine T.

Some studies have indicated the interference of TAG on AOPP levels [29, 30]. The effect of TAG concentration acquires significance in hypertriglyceridemic state associated with diabetes, atherosclerosis, and nephropathy [29, 30]. In the present study, the spectrophotometric measurement of AOPP concentration based on reaction with chloramine T does not eliminate the interference of TAG. Probably, positive and significant correlations in the AAA group for AOPP and TAG are the effect of high level of TAG on AOPP concentration.

Promotion of oxidative stress and inflammation leads to the acceleration of atherosclerosis as a result of accumulation of AOPP and C=O [31, 32]. The relatively long half-lives of modified proteins make them attractive biomarkers of oxidative stress [30, 33]. Peters points that half-life of albumin, the main source of AOPP in plasma, is about 19 days [34]. However, many oxidative modifications of proteins generate irreversible and long half-life products which are not rapidly degraded [23]. For that reason, protein-bound di-Tyr is classified as chemically stable product and long-lasting biomarker of oxidative stress in patients with end-stage renal disease (ESRD) [34]. On the other hand, the degradation pathway of oxidatively modified proteins has not yet been precisely defined. Marotta et al. demonstrated that glycated proteins (AGE) are less subject to digestion by proteolytic enzymes [35]. Furthermore, they also hypothesized that significantly changed structure of AGE in comparison with unglycated physiological protein is not recognized by the active site of digestion enzymes, which may result in deterioration in their excretion and accumulation [35]. In addition, participation of the proteasome system in degradation of irreversibly modified proteins, such as those containing C=O groups, was many times discussed [36, 37]. It was proposed that the proteasome is able to recognize and degrade oxidized proteins. However, this pathway is proposed for living cells not for circulating blood plasma proteins. In this regard, we can hypothesize that significantly higher concentration of AOPP in HD group in comparison with PRE is the result of accumulation of modified proteins. This is the result more in deterioration in their degradation than in renal failure. In our opinion, chronic renal failure increases oxidative stress, especially carbonyl stress, as a result of the disturbed elimination of uric toxins.

On the basis of our results, we can conclude that types of atherosclerosis, “classic” or “nonclassic,” associated with AAA, AIOD, and CKD and, consequently, theirs accompanying oxidative stress have a different impact on modification of proteins. Furthermore, the spectrophotometric methods do not reflect the total concentration of AOPP and carbonylated proteins. Therefore, further studies are necessary in order to conclude a main pathway of protein modification with reference to AAA, AIOD, and CKD. It may be suggested that rather more advanced modifications of proteins take place in PRE and HD patients than AAA and AIOD.

Additionally, in AAA group, significant and positive correlation was found between AOPP and FB which led us to conclude that AOPP are derived from FB as well as albumin. In the present study, by dividing PRE group into quartiles according to AOPP concentration, a relationship between AOPP and WBC count was revealed. The highest WBC was observed in IV quartile, and it may be suggested that patients with the highest AOPP concentration are characterized by more intense inflammation in comparison to patients in lower AOPP quartiles. We found the highest concentrations of hsCRP and WBC count, but lower UA level in AIOD. This suggests that inflammation that accompanies AIOD is probably more intensified and results in an increased oxidative stress.

The concentration of ROS is regulated in vivo by the antioxidant defense system including enzymes such as superoxide dismutase, ceruloplasmin, paraoxonase, and catalase [13, 38–41]. The same role is performed by albumin, bilirubin, uric acid, or exogenous antioxidants such as vitamins (A, E, C, and *β*-carotene) and polyphenolic compounds [39–41]. In the present study, UA as a representative of endogenous antioxidant defense system was determined. Interestingly, significantly lower UA and higher concentration of WBC were observed in AIOD group. Moreover, in our study, significantly higher concentration of UA was demonstrated in AAA group in comparison to AIOD. This may suggests that oxidative stress which accompanies AIOD progression is probably more intensified in comparison with AAA and results in an increased antioxidant defense system mobilization.

## 5. Study Limitations and Strengths

The main quality of the present study is establishing the effect of three different atherosclerosis-mediated diseases (AAA, AIOD, and CKD) on the selected modification of proteins. Based on literature data, oxidative modification of proteins and the products of this modification have been thus far poorly investigated in reference to AAA and AIOD, especially. On the contrary, lipid peroxidation products have been repeatedly estimated and discussed in association with many pathological conditions, including AAA, AIOD, and CKD. The study was performed in rather small groups, especially AIOD (*n* = 13), and containing different numbers of men and women. However, it should be stressed that, despite the small sizes of the groups, their biochemical and clinical characteristics well match the specifics of the diseases. Notwithstanding this fact, the results obtained ex vivo are consistent. On the basis of the obtained results, we confirmed that both vascular disease and chronic kidney disease contribute to modification of proteins. Furthermore, AAA, AIOD, and CKD-related atherosclerosis promote modification of proteins in different ways. However, the spectrophotometric methods applied in the present study seem to be limitation because they do not reflect the total AOPP and C=O content in the studied groups. The main pathway of protein modification with reference to AAA, AIOD, and CKD is still unknown, but we can assume that more advanced modifications of proteins take place in CKD (PRE and HD groups) than among AAA or AIOD patients. However, larger and homogenous population-based studies and more advanced analytical methods are needed to confirm the above-mentioned results.

## 6. Conclusions

Vascular disease, AIOD and AAA, and chronic kidney disease, PRE and HD, contribute to formation of advanced oxidation protein products and carbonylation of proteins. AAA, AIOD, and CKD-related atherosclerosis promote modification of proteins in different way. Similar levels of C=O in HD and PRE patients suggest that carbonylation of plasma proteins, which reflect “carbonyl stress,” accompany CKD-related atherosclerosis. In both AAA and AIOD patients, similar C=O and AOPP levels suggest that “classic atherosclerosis” and its oxidative stress influence plasma proteins.

In AIOD, positive correlation of AOPP and C=O and negative correlation of U and both AOPP and C=O, higher hsCRP, FB, and WBC levels suggest that inflammation accompanying AIOD is probably more intense and results in an increased oxidative stress. Furthermore, the spectrophotometric methods do not reflect the total concentration of AOPP and carbonylated proteins. It may be suggested that more advanced modifications of proteins take place rather in PRE and HD patients than AAA and AIOD.

## Figures and Tables

**Table 1:** Clinical characteristics of analyzed groups of patients with AAA, AIOD, PRE, and HD.

Parameters	AAA (35 patients) number (%)	AIOD (13 patients) number (%)	PRE (50 patients) number (%)	HD (34 patients) number (%)
Age (mean ± SD)	69.97 ± 9.97	63.54 ± 7.55	71.6 ± 13.12	54.03 ± 16.18
Gender (male/female)	26/9	9/4	27/23	23/11
Hypertension	30 (85.7)	8 (61.5)	50 (100)	34 (100)
Hypercholesterolemia	7 (20)	3 (23)	50 (100)	34 (100)
Coronary artery disease	19 (54.3)	7 (53.8)	17 (34)	34 (100)
Previous myocardial infarction	6 (17)	2 (15.4)	8 (16)	9 (26.5)
Cerebrovascular accident	5 (14.3)	—	1 (2)	4 (11.8)
Kidney disease	6 (17)	2 (15.4)	50 (100)	34 (100)
Pulmonary disease	4 (11.4)	2 (15.4)	0	0

Medications				

*β*-Blocker	17 (48.6)	7 (53.8)	29 (58)	20 (58.8)
ACEI	18 (51.4)	5 (38.5)	25 (50)	11 (32)
Statins	18 (51.4)	10 (77)	39 (78)	27 (79.4)
NSAID	35 (100)	13 (100)	40 (80)	11 (32.4)

**Table 2:** Biochemical parameters in the plasma of AAA, AIOD, PRE, and HD patients.

Parameters	AAA	AIOD	PRE	HD
Total cholesterol (TC) [mmol/L]	4.45 ± 1.37	4.31 ± 1.85	5.02 ± 2.23	4.30 ± 1.16
LDL-cholesterol (LDL-C) [mmol/L]	2.44 ± 1.16	2.81 ± 1.41	3.83 ± 2.08	2.75 ± 0.57
HDL-cholesterol (HDL-C) [mmol/L]	1.30 ± 0.51	1.13 ± 0.26	1.63 ± 0.44^d^	1.21 ± 0.23
Triacylglycerols (TAG) [mmol/L]	1.54 ± 0.85	1.35 ± 0.57	1.58 ± 0.90	1.66 ± 0.51
Uric acid (UA) [*μ*mol/L]	376.68 ± 96.27	305.77 ± 101.75^a^	405.30 ± 107.20^d^	367.76 ± 107.85
Red blood cells (RBC) [10^12^/L]	4.61 ± 0.45	4.81 ± 0.76	3.65 ± 0.60^bd^	3.48 ± 0.43^ce^
White blood cells (WBC) [10^9^/L]	7.77 ± 2.57	9.60 ± 2.89^a^	6.72 ± 2.19^bd^	6.44 ± 1.60^ce^
Hematocrit (HCT) [L/L]	0.41 ± 0.04	0.43 ± 0.06	0.32 ± 0.03^bd^	0.32 ± 0.03^ce^
Platelets (PLT) [10^9^/L]	251.60 ± 101.29	284.77 ± 126.90	232.80 ± 109.90	252.83 ± 105.20
Hemoglobin (HGB) [mmol/L]	8.41 ± 0.93	8.86 ± 1.18	6.85 ± 0.71^bd^	6.83 ± 0.68^ce^
Fibrinogen (FB) [mg/dL]	325.22 ± 92.57	349.44 ± 129.70	NA	NA
Creatinine (Cr) [*μ*mol/L]	107.54 ± 60.87	87.31 ± 21.55	217.00 ± 91.48^bd^	736.50 ± 241.70^cef^
Urea (U) [mmol/L]	6.77 ± 3.59	5.12 ± 1.72	15.33 ± 5.23^bd^	15.30 ± 7.16^ce^
eGFR [ml/min/1.73 m^2^]	61.88 ± 18.00	70.4 ± 13.5	25.05 ± 10.70^bd^	7.29 ± 3.18^cef^
hsCRP [mg/L]	8.53 ± 5.73	6.64 ± 4.78	9.90 ± 5.35	4.25 ± 3.18^cf^
Albumin [g/L]	37.24 ± 4.68	37.08 ± 5.03	38.20 ± 6.90	36.90 ± 12.48
AOPP [*μ*mol/mg protein]	5.20 ± 2.00	4.33 ± 1.77	1.13 ± 0.42^bd^	1.74 ± 0.70^cef^
C=O [nmol/mg protein]	1.10 ± 0.80	0.96 ± 0.50	5.66 ± 2.85^bd^	4.83 ± 2.09^ce^

All data are expressed as mean ± standard deviation; NA: not analyzed; significant differences: ^a^AAA versus AIOD; ^b^AAA versus PRE; ^c^AAA versus HD; ^d^AIOD versus PRE; ^e^AIOD versus HD; ^f^PRE versus HD *p* ≤ 0.05.

**Table 3:** The correlations coefficients for AOPP and C=O in study groups of patients.

	*r*	Group of patients
AOPP^*∗*^		
C=O	0.6158	AIOD
TC	0.4180	AAA
LDL-C	0.3964	AAA
TAG	0.3814	AAA
FB	0.5046	AAA
U	−0.5952	AIOD

C=O^*∗*^		
eGFR	−0.3848	AAA
U	−0.6664	AIOD
UA	0.4588	AAA
HCT	0.2839	PRE
WBC	0.3753	HD

*r*: correlation coefficient ^**∗**^*p* ≤ 0.05.

**Table 4:** Comparison between patients categorized into quartiles according to AOPP and C=O concentration.

Parameter	Quartile I	Quartiles II and III	Quartile IV
AAA			

	AOPP < 3.759	AOPP 3.759–6.542	AOPP > 6.542

TC	3.514 ± 0.886	4.52 ± 0.935^*∗*^	4.973 ± 2.371
TAG	0.885 ± 0.467	1.711 ± 0.889^*∗*^	1.399 ± 0.624
FB	265.3 ± 39.84	341.1 ± 103.3	345.0 ± 81.09^*∗*^

AIOD			

	AOPP < 2.888	AOPP 2.888–5.872	AOPP > 5.872

C=O	0.552 ± 0.314	0.902 ± 0.140^*∗*^	1.503 ± 0.987
U	7.217 ± 1.432	4.599 ± 1.349^*∗*^	4.013 ± 1.129^*∗*^

	C=O < 0.70	C=O 0.70–1.04	C=O > 1.04

AOPP	3.068 ± 0.648	3.887 ± 1.655	6.827 ± 0.824^*∗*^

PRE			

	AOPP < 0.902	AOPP 0.902–1.204	AOPP > 1.204

WBC	6.715 ± 1.991	6.130 ± 1.900	7.998 ± 2.575^*∗∗*^
eGFR	31.08 ± 14.20	22.49 ± 8.49^*∗*^	24.20 ± 8.85

HD			

	C=O < 3.50	C=O 3.50–5.23	C=O > 5.23

AOPP	2.117 ± 0.654	1.593 ± 0.699^*∗*^	1.687 ± 0.622

^*∗*^Differences versus quartile I *p* ≤ 0.05; ^*∗∗*^differences versus quartiles II and III *p* ≤ 0.05.
